# Factors associated with palliative care attitudes among undergraduate nursing students through personal experiences: A cross-sectional study

**DOI:** 10.1371/journal.pone.0355951

**Published:** 2026-08-14

**Authors:** Yang Xie, Qingyan Chen, Jia Xiao, Chunyan Li

**Affiliations:** Nursing Major, School of Nursing, Hunan University of Chinese Medicine, Changsha, Hunan, China; Universitas Pendidikan Ganesha, INDONESIA

## Abstract

**Objective:**

This study aims to analyze the factors associated with the personal experiences of undergraduate nursing students regarding their attitudes toward palliative care, thereby providing evidence for targeted humanities education in nursing.

**Methods:**

A cross-sectional survey was conducted among 467 undergraduate nursing students between December 2024 and March 2025 using convenience sampling. Data were collected using a general information questionnaire and the Chinese version of the Frommelt Attitudes Toward Care of the Dying Scale (FATCOD-B-C). Statistical analyses included independent sample t-tests, one-way ANOVA, and multiple linear regression.

**Results:**

The mean total score of the FATCOD-B-C among undergraduate nursing students was 96.75 ± 8.80, indicating a moderately high level of attitudes toward palliative care. Multiple regression analysis revealed that female gender and a family member with a terminal illness were positively associated with palliative care. Conversely, being in a higher academic year, holding a neutral attitude toward the nursing profession, lacking hospital internship experience, previous bereavement experience, caring for a dying patient, and having no previous lectures and training in palliative care as factors negatively associated with palliative care.

**Conclusion:**

Undergraduate nursing students generally exhibit a positive attitude toward palliative care; however, personal experiences have a multifaceted impact on their attitudes. Educators should focus on cultivating students’ professional identity and enhancing positive experiences such as clinical internships. Additionally, attention should be given to the emotional needs of students in higher academic years and those who have experienced bereavement, with an emphasis on strengthening death education and experiential learning to develop more positive attitudes toward palliative care.

## Introduction

Globally, as population aging continues to accelerate and the disease spectrum shifts toward chronic non-communicable diseases, providing dignified and high-quality end-of-life care for patients at the end of life has become an urgent public health and social issue [1]. Internationally, there have been calls to scale up palliative care services, such as World Health Assembly Resolution 67.19, which designates palliative care as an essential component of universal health coverage. Based on projected annual increases of 3.9% in incidence and 2.5% in mortality, cancer-related deaths in mainland China are projected to exceed 4.5 million and 3.62 million, respectively, by 2030 [2]. However, currently, only about 14% of the global need for palliative care is met [3], while the demand for palliative care services is increasing [4]. The end-of-life stage of human life is often accompanied by complex physical symptoms, psychological distress, social challenges, and spiritual needs, where the traditional curative-oriented medical model often reveals its limitations [5]. These complex health challenges highlight the essentiality of developing palliative care that is patient-centered and aimed at enhancing the quality of life at the end of life.

As a vital component of the healthcare system, palliative care aims to alleviate the suffering of terminal patients, safeguard their dignity, and provide comprehensive physical, psychological, social, and spiritual support for both patients and their families. With the acceleration of population aging and the shifting disease spectrum, the demand for high-quality palliative care is increasing, requiring that future primary healthcare providers—particularly nursing students—possess professional knowledge and cultivate positive attitudes toward end-of-life care [6–8]. Attitude, as a stable psychological tendency held by an individual toward a specific object, encompasses three dimensions: cognitive, affective, and behavioral intentions, and serves as a key predictor of their future clinical practice behaviors [9]. However, palliative care education in China started relatively late, and the educational system remains underdeveloped, with insufficient emphasis placed on palliative care education for nursing students, which contributes to nursing students generally holding negative attitudes toward engaging in palliative care [10], with palliative care nursing ranking among the least desirable career choices for nursing students [11]. Research indicates that the attitudes of healthcare professionals, including students still in training, toward palliative care directly impact their willingness to provide such care, the quality of care delivered, and the effectiveness of communication with patients and their families [12]. However, compared with relatively objective knowledge and skills, the formation and shaping mechanism of attitudes is more complex, as it is deeply embedded in individuals’ values, sociocultural backgrounds, and crucially, personal experiences [9].

Personal experiences are widely recognized as a foundational force in shaping individuals’ attitudes, beliefs, and even worldviews [13]. Research indicates that direct, concrete personal experiences can be more profoundly and persistently associated with changes in an individual’s attitude system, providing unique insights and support based on personal experiences [14]. Previous studies have shown that authentic clinical experiences contribute to the development of positive attitudes toward death among nursing students and enhance their willingness to provide palliative care [15]. For nursing students, personal and professional experiences may play a more decisive role than their education in shaping their attitudes and behaviors toward palliative care [16]. The composition of their personal experiences encompasses not only the life course common to all individuals but also the uniqueness conferred by their professional training pathway [13]. During the professional education phase, students, as “quasi-professionals,” have their initial exposure to and engagement in the management of illness, suffering, and death—for example, through history of contact with terminally ill patients [17], witnessing death or caring for a dying patient [15,18], or previous bereavement experience [16,19,20]. These experiences, which are emotionally and cognitively challenging, can significantly be associated with nursing students’ attitudes toward palliative care. Additionally, medical-related social practice and volunteer activities (e.g., serving in nursing homes or palliative care institutions), provide a window for observing the end of life in different contexts, with such experiences enhancing students’ understanding of palliative care and facilitating attitudinal shifts [21]. More profound are health crises involving oneself or close relatives, such as personal experiences with serious illness or having a family member diagnosed with a major disease [15]. This “role transition” may greatly enhance empathetic ability. Experiences directly related to death, such as experiences of dealing with dead patients [22] or attending funerals [15] exert a particularly strong impact on students’ attitudes toward death due to their highly symbolic nature. Meanwhile, whether students have actively sought understanding or educational experiences in palliative care [19] as a form of cognitive preparation is also significantly associated with their professional identity. Domestic studies have predominantly focused on students’ general death anxiety or knowledge levels, yet there has been limited research on the inherent attitudinal differences arising from personal experiences and practical exposure. The exploration of “personally relevant experiences” as a key variable associated with nursing students’ attitudes toward palliative care may have been insufficiently addressed.

Therefore, grounded in Social Cognitive Theory and Experiential Learning Theory, and responding to the urgent global need to improve the quality of end-of-life care, this study was designed to systematically examine, through a cross-sectional survey, the association between personally relevant experiences and attitudes toward palliative care among nursing students. By elucidating the specific relationships between personal experiences and attitudes, we aim to provide an empirical basis for developing more targeted and empathetic nursing humanities education and palliative care training. This will ultimately contribute to cultivating future nursing professionals who are not only responsive to international care trends but also possess both professional competence and humanistic compassion.

## Methods

### Study design and sample size

The primary outcome measure of this study is the total score of the FATCOD-B-C scale, which is a continuous variable. Therefore, the sample size calculation formula for continuous variables in cross-sectional studies was used:


$$\begin{array}{c} n=\;\frac{{\left(Z_{\frac{\alpha }{\text{2}}}\;+\;Z_{\beta }\right)}^{\text{2}}\cdot \;\sigma^{\text{2}}}{\delta^{\text{2}}} \end{array}$$ (1)

The two-sided significance level was set at α = 0.05 (Z_α/2_ = 1.96), and the power was set at 1-β = 0.80 (Z_β_ = 0.84). The standard deviation σ was based on the total score of the FATCOD-B-C scale among Chinese nursing students reported by Liu Yang et al. [23] (σ = 10.68). Regarding the determination of effect size, this study adopted the minimum effect size criterion (Cohen’s d = 0.2). Accordingly, a minimum of 196 participants was required. Considering a 20% non-response rate, the final required sample size was 245 participants.

### Participants and period

In this study, convenience sampling was used to recruit participants from a university in Hunan Province, China. Inclusion criteria were: full-time undergraduate nursing students; those who provided informed consent and voluntarily participated in the survey; and those who could correctly understand the questionnaire content without reading difficulties. Students who voluntarily withdrew from the survey during the process for various reasons were excluded. The recruitment process was conducted through the university’s online learning platform and class-based WeChat groups. The researchers posted an invitation letter containing a brief introduction to the study purpose along with a voluntary participation link. Interested students voluntarily clicked the link and completed the online questionnaire after selecting ‘agree to participate in this study’ on the informed consent page on the first page of the questionnaire. A total of 493 undergraduate nursing students were recruited to participate in this study between December 2024 and March 2025.

### Measurements

Based on a literature review and expert consultation, a self-designed general information and demographic questionnaire was developed, including items on gender, age, nationality, religious belief, residence, annual family income, self-perceived health status, grade, and attitude toward the nursing major. Based on previous studies, 10 questions related to palliative care experiences were formulated, comprising: hospital internship experience, experience of participating in medical-related voluntary service, experience of suffering from serious illness, a family member with a terminal illness, previous bereavement experience, history of contact with terminally ill patients, experience of dealing with dead patients, attendance at funerals, cared for a dying patient, previous lectures & training in palliative care.

The Chinese version of the Frommelt Attitudes Toward Care of the Dying Scale (FATCOD), originally developed by Dr. Frommelt in the United States starting in 1989 [24], is used to measure nurses’ attitudes toward end-of-life care and consists of 30 items. The Chinese version of the Frommelt Attitudes Toward Care of the Dying Scale, Form B (FATCOD-B-C), was subsequently localized and revised by Dr. Wang Liping [25]. This version comprises 29 items and demonstrates good reliability and validity. The scale demonstrates good internal consistency (Cronbach’s α = 0.796 for the total scale; Cronbach’s α for subscales ranges from 0.610 to 0.863), satisfactory test-retest reliability (r = 0.959, *P* < 0.001), and high content validity (content validity index = 0.92). This scale has been used multiple times and repeatedly validated in Chinese nursing student populations [23,26]. Items are scored on a 5-point Likert scale (coded as follows: 1 = “strongly disagree,” 2 = “disagree,” 3 = “uncertain,” 4 = “agree,” 5 = “strongly agree”), with total scores ranging from 29 to 145. Positively worded items (1, 2, 4, 16, 18, 20, 21, 22, 23, 24, 25, 27, and 30) are scored directly from 1 to 5. The remaining items are reverse-coded—i.e., ‘strongly disagree’ (1) is recoded to 5, and ‘strongly agree’ (5) is recoded to 1—before calculating the total score.

In this study, when percentage agreement is reported for selected Likert scale items, ‘agree’ was defined as the proportion of participants who selected either ‘agree’ (4) or ‘strongly agree’ (5) on the 5-point scale. Neutral responses (3) were not included in the agreement percentage. ‘Disagree’ was defined as selecting ‘disagree’ (2) or ‘strongly disagree’ (1). Higher scores indicate more positive attitudes. In this study, the Cronbach’s α coefficient for the total scale was 0.914.

### Ethical considerations and procedures

This study maintains the anonymity of all the subjects, who can opt whether or not to participate. The research group of this study further guaranteed that the identity of the subjects would not be disclosed and that their answers would be confidential. Ethical approval for the project was gained from the Science and Technology Ethics Review Committee of Nursing and Behavioral Medicine, Hunan University of Chinese Medicine [grant numbers ZYYHLLL-2024-03-007].

To facilitate data collection, electronic questionnaires were distributed using Wenjuanxing, an online survey platform. The questionnaire link was shared via WeChat with nursing students. The distributors of the questionnaire, comprising faculty members from the nursing schools of various universities, received unified training. Participants were required to read the informed consent form before answering the research questions. Informed consent was obtained electronically. After clicking the questionnaire link, participants first entered an independent informed consent page that provided a detailed explanation of the study’s purpose, content, voluntary participation principle, confidentiality assurance, and the right to withdraw from the study at any time. At the bottom of the page, there was a checkbox labeled ‘I have read and understood the above information and agree to participate in this study,’ along with an ‘I do not agree’ option. Only after participants actively checked the ‘agree’ box could they proceed to complete the formal questionnaire. Participants who selected ‘I do not agree’ were automatically redirected to the end of the questionnaire and exited. All participants in this study completed the above informed consent procedure. After excluding invalid questionnaires—those with a completion time of less than one minute and those with duplicate selections—a total of 467 valid questionnaires were included, yielding an effective response rate of 94.7%.

### Data analysis

Data analysis was performed using the SPSS statistical software package (Version 27). To ensure the accuracy of data entry and analysis, two individuals independently conducted the data organization and statistical procedures. Frequencies were reported as n (%) Descriptive statistics were used to report demographics and attitudes toward palliative care. Independent samples t-tests and one-way ANOVA were employed to compare differences in palliative care attitudes across demographic variables. Statistically significant variables were entered into multiple linear regression. Candidate variables for the multiple linear regression model were selected based on bivariate analyses, prior literature, and theoretical considerations. Guided by Social Cognitive Theory [27] and informed by previous studies on nursing students’ attitudes toward end-of-life care [15,19], we identified three domains of potential predictors: demographic characteristics, personal experiences, and cognitive preparation. All candidate variables were examined for multicollinearity prior to model entry using the variance inflation factor (VIF), with a threshold of VIF < 10. The final model was constructed using the Enter method, in which all selected variables were simultaneously entered into the model. Multiple linear regression analysis was conducted to identify factors associated with attitudes toward palliative care. The test level α = 0.05 and *P* < 0.05 indicated significant differences.

## Results

### Sample characteristics

A total of 467 potentially eligible participants were included in this study, with no missing values. The age of participants was mostly concentrated between 18 and 20 years (19.36 ± 1.51). Among them, 361 participants were female (77.30%), and 106 were male (22.70%). Most students held a neutral attitude toward the nursing profession (70.00%). In terms of personal experiences, 88 participants (18.80%) had hospital internship experience, while 210 participants (45.00%) had no experience of losing a relative. The vast majority of students reported no experience caring for dying patients (91.90%). Regarding whether they had received lectures or training in palliative care, 48.80% answered ‘yes’ and 51.20% answered ‘no.’ Detailed demographic data are presented in Table 1.

**Table 1:** Demographic and experiential characteristics of nursing students (N = 467).

Students’ characteristics	n(%)	The Attitude score of FATCOD-B-C	*t/F*	*P*
**Gender**				
Male	106(22.70)	94.32 ± 8.47	−3.264	0.001
Female	361(77.30)	97.46 ± 8.77		
**Age**				
18-20	369(79.00)	96.69 ± 8.60	−0.294	0.769
21-23	98(21.00)	96.98 ± 9.53		
**Nationality**				
The Han nationality	414(88.70)	96.85 ± 8.65	−0.706	0.48
Minority	53(11.30)	95.94 ± 9.89		
**Religious belief**				
No	448(95.90)	96.87 ± 8.82	1.472	0.142
Yes	19(4.10)	93.84 ± 7.75		
**Residence**				
Province-level region	57(12.20)	95.67 ± 9.10	0.524	0.666
Prefecture-level region	61(13.10)	96.28 ± 8.34		
Town	134(28.70)	97.30 ± 8.88		
Rural area	215(46.00)	96.82 ± 8.82		
**Annual family income (RMB)**				
<50000	221(47.30)	95.98 ± 8.71	1.129	0.337
50000-100000	161(34.50)	97.51 ± 8.44		
100000-300000	75(16.10)	97.44 ± 9.02		
>300000	10(2.10)	96.2 ± 13.60		
**Self-rated health status**				
Good	325(69.60)	96.77 ± 8.61	0.008	0.992
General	123(26.30)	96.72 ± 9.42		
Unhealthy	19(4.10)	96.53 ± 8.13		
**Grade**				
Freshman	180(38.50)	97.24 ± 8.54	1.176	0.319
Sophomore	100(21.40)	95.32 ± 8.12		
Junior	110(23.60)	97.23 ± 8.74		
Senior	77(16.50)	96.77 ± 10.18		
**Attitude toward nursing major**				
Positive	105(22.50)	98.33 ± 9.34	2.547	0.079
Neutral	327(70.00)	96.41 ± 8.54		
Negative	35(7.50)	95.14 ± 9.09		
**Personal experiences**				
**Hospital internship experience** ^**1**^				
Yes	88(18.80)	97.32 ± 10.11	0.605	0.546
No	379(81.20)	96.62 ± 8.47		
**Experience of participating in medical related voluntary service**				
No	278(59.50)	96.49 ± 8.36	−0.752	0.453
Yes	189(40.50)	97.13 ± 9.41		
**Experience of suffering from serious illness**				
No	429(91.90)	96.67 ± 8.63	−0.627	0.531
Yes	38(8.10)	97.61 ± 10.57		
**A family member with a terminal illness**				
No	319(68.30)	96.37 ± 8.74	−1.363	0.147
Yes	148(31.70)	97.56 ± 8.89		
**Previous bereavement experience**				
No	210(45.00)	97.57 ± 9.48	1.835	0.067
Yes	257(55.00)	96.07 ± 8.16		
**History of contact with terminally ill patients**				
No	328(70.20)	96.65 ± 8.60	−0.369	0.712
Yes	139(29.80)	96.98 ± 9.27		
**Experience of dealing with dead patients**				
No	414(88.70)	96.96 ± 8.90	1.438	0.151
Yes	53(11.30)	95.11 ± 7.85		
**Attendance at funerals**				
No	82(17.60)	96.68 ± 11.40	0.059	0.953
Yes	385(82.40)	96.76 ± 8.15		
**Cared for a dying patient** ^**2**^				
No	429(91.90)	97.01 ± 8.80	2.21	0.028
Yes	38(8.10)	93.74 ± 8.26		
**Previous lectures and training in palliative care** ^**3**^				
Yes	228(48.80)	98.50 ± 8.93	4.294	<0.001
No	239(51.20)	95.07 ± 8.34		

^1^clinical internship experience in a general hospital.

^2^This variable refers to direct participation in the care of dying patients, either in clinical internships or in daily life, including nursing behaviors such as vital sign monitoring, symptom management, and psychological support. It also includes personal care provided to family members or relatives at home.

^3^whether participants had received at least one specialized lecture or training session on palliative care, with a duration of 30 minutes.

### Attitude toward palliative care

The overall mean score of undergraduate nursing students on the Chinese version of the Frommelt Attitudes Toward Care of the Dying Scale, Form B (FATCOD-B-C) was 96.75 ± 8.80, indicating a moderately positive attitude. In terms of attitude scores, the difference between male and female students was statistically significant (t = −3.264, *P* = 0.001), with female students scoring higher (97.46 ± 8.77) than male students (94.32 ± 8.47). However, the overall difference in attitude scores across different grades was not statistically significant (*F* = 1.176, *P* = 0.316). Students who had never cared for a dying patient scored significantly higher than those who had experience caring for a dying patient (t = 2.21, *P* = 0.028). Additionally, students who had previous lectures or training in palliative care scored significantly higher than those without such experience (t = 4.294, *P* < 0.001).

By comparing these items, it can be seen that: “Recognition of the value of caring for dying patients. Students showed strong agreement with items reflecting the meaningfulness of end-of-life care, such as “Giving care to the dying person is a worthwhile experience” (4.51 ± 0.78) and “Families should be concerned about helping their dying member make the best of his/her remaining life” (4.20 ± 0.83).

Positive attitudes toward family involvement. Students endorsed items supporting family participation in care, including “Caring for the patient’s family should continue throughout the period of grief and bereavement” (4.24 ± 0.89) and “The family should be involved in the physical care of the dying person if they want to” (4.09 ± 0.82).

Acknowledgment of death and emotional preparation. Moderately high scores were observed for items such as “Death is not the worst thing that can happen to a person” (4.14 ± 0.94) and “It is beneficial for the dying person to verbalize his/her feelings” (3.99 ± 0.87), indicating a degree of acceptance and emotional openness (Table 2).

**Table 2:** The results of the Chinese version of FATCOD-B-C (N = 467). *Reverse scored.

Item	Mean (SD)
1. Giving care to the dying person is a worthwhile experience.	4.51 ± 0.78
2. Death is not the worst thing that can happen to a person.	4.14 ± 0.94
3. I would be uncomfortable talking about impending death with the dying person. ^*^	2.61 ± 1.16
4. Caring for the patient’s family should continue throughout the period of grief and bereavement.	4.24 ± 0.89
5. I would not want to care for a dying person. ^*^	3.26 ± 1.09
12. The family should be involved in the physical care of the dying person if they want to.^*^	4.09 ± 0.82
16. Families need emotional support to accept the behavior changes of the dying person.	3.94 ± 0.88
17. As a patient nears death, the nonfamily caregiver should withdraw his/her involvement with the patient.^*^	1.88 ± 0.87
18. Families should be concerned about helping their dying member make the best of his/her remaining life.	4.20 ± 0.83
20. Families should maintain as normal an environment as possible for their dying member.	4.02 ± 0.85
21. It is beneficial for the dying person to verbalize his/her feelings.	3.99 ± 0.87
30. It is possible for non-family care-givers to help patients prepare for death.	3.91 ± 1.01

### Attitudes toward palliative care and associated factors

The total score of the FATCOD-B-C scale was taken as the dependent variable, and factors identified as potential predictors based on bivariate analyses, prior literature, and theoretical considerations were entered as independent variables. Multiple linear regression analysis was conducted using the Enter method, with a threshold of α = 0.05 for statistical significance. The specific variables ultimately included in the regression model, along with their coding and assignment details, are presented in Table 3.

**Table 3:** Assignment Table.

Correlation variables	Assignment Instructions
Gender	Male = 1; Female = 2
Age	18-20 = 1; 21-23 = 2
Nationality	The Han nationality = 1; Minority = 2
Religious belief	No = 1; Yes = 2
Self-rated health status	Good = 1; General = 2; Unhealthy = 3
Grade	Freshman = 1; Sophomore = 2; Junior = 3; Senior = 4
Attitude toward the nursing major	Positive = 1; Neutral = 2; Negative = 3
**Personal experiences**	
Hospital internship experience	Yes = 1; No = 2
Experience of participating in medical related voluntary service	No = 1; Yes = 2
Experience of suffering from serious illness	No = 1; Yes = 2
A family member with a terminal illness	No = 1; Yes = 2
Previous bereavement experience	No = 1; Yes = 2
History of contact with terminally ill patients	No = 1; Yes = 2
Experience of dealing with dead patients	No = 1; Yes = 2
Attendance at funerals	No = 1; Yes = 2
Cared for a dying patient	No = 1; Yes = 2
Previous lectures and training in palliative care	Yes = 1; No = 2

In the multiple linear regression analysis, female gender (*B =* 3.295*, P* < 0.001) and having a family member with a terminal illness (*B* = 1.901*, P* = 0.038) were identified as positive factors associated with undergraduate nursing students’ attitudes toward palliative care. Conversely, a lack of hospital internship experience (*B* = −5.362*, P* < 0.05), being in a higher academic year (*B* = −8.626*, P* = 0.017), a neutral attitude toward the nursing profession (*B* = −1.980*, P* < 0.05), having previous bereavement experience (*B* = −1.787*, P* = 0.042), and having experience caring for a dying patient (*B =* −3.461*, P* = 0.039) were identified as negative factors associated with attitudes toward palliative care (Table 4). This study found that participants without internship experience had significantly lower attitude scores (*B* = −5.362, *P* < 0.05). Nursing students with internship experience exhibited more positive attitudes toward palliative care. The regression analysis further confirmed that internship experience is an independent positive predictor of attitude scores. The adjusted R² was 0.084, indicating that the included independent variables explained only 8.4% of the total variance in attitudes toward palliative care.

**Table 4:** Multiple Linear Regression Analysis of FATCOD-B-C Scores among Undergraduate Nursing Students (N = 467).

Variable	B	SE	β	t	*P*	95% CI	
						Lower bound	Upper bound
Constant	110.782	6.774	–	16.354	<.001	97.469	124.096
Gender = Female (Ref: Male)	3.295	0.974	0.157	3.382	<.001	1.380	5.210
Grade = Sophomore (Ref: Freshman)	−2.233	1.124	−0.104	−1.988	.047	−4.442	−0.025
Grade = Junior (Ref: Freshman)	−3.037	1.29	−0.147	−2.355	.019	−5.572	−0.503
Grade = Senior (Ref: Freshman)	−8.626	3.591	−0.364	−2.402	.017	−15.684	−1.568
Attitude toward nursing major = Neutral (Ref: Positive)	−1.980	1.003	−0.103	−1.975	.049	−3.951	−0.010
Hospital internship experience (Ref: Yes)	−5.362	2.713	−0.239	−1.976	.049	−10.695	−0.030
A family member with a terminal illness (Ref: No)	1.901	0.912	0.101	2.084	.038	0.108	3.693
Previous bereavement experience (Ref: No)	−1.787	0.875	−0.101	−2.042	.042	−3.507	−0.067
Cared for a dying patient (Ref: No)	−3.461	1.672	−0.108	−2.07	.039	−6.747	−0.175
Previous lectures and training in palliative care (Ref: Yes)	−3.758	0.923	−0.214	−4.072	<.001	−5.572	−1.944

Note: *B*, regression coefficient; *SE, standard error; Beta, standardized* coefficient Beta.

F = 2.581, p <.001, R = 0.370, coefficient of determination (R^2^) = 0.137, adjusted R^2^= 0.084.

## Discussion

This study indicates that undergraduate nursing students hold a positive attitude toward palliative care, with attitude scores being similar to those reported by Delgersuren Gelegjamts et al. (96.37 ± 7.59) [18], but lower than those of nursing students in Vietnam (103.58 ± 7.21) [28], Italy (115.3 ± 9.1) [20], and Switzerland (117.7 ± 9.8) [17]. However, when interpreting this cross-country comparison, differences in palliative care education systems and cultural backgrounds across countries need to be considered. According to the recommendations issued by the European Association for Palliative Care (EAPC), palliative care content was integrated into the medical curricula of many European countries years ago [29]. For example, medical students at the University of Basel are required to complete up to 5 hours of palliative care education, and palliative care has become an important component of universal health coverage [30]. Compared with Western developed countries, palliative care education in China may still be in its early stages [10], and the emphasis placed on palliative care education for nursing students may be insufficient. In contrast, in Arab cultures, religious individuals tend to hold more inclusive attitudes toward dying patients, stemming from their beliefs in the afterlife [31]. However, China has long-standing cultural taboos surrounding death and dying, where open discussion is often considered impolite or even forbidden [32,33]. This perspective is directly supported by data from the present study: 48.20% of students agreed with item 3, ‘Talking about impending death with dying patients makes me feel uncomfortable’; 35.50% agreed with item 6, ‘Healthcare professionals should avoid discussing death with dying patients’; and 33.00% explicitly agreed with item 13, ‘If the patient I am caring for dies, I hope I am not present ‘ (see Table 2). These responses reflect a tendency associated with Confucian values [34]—emphasizing ‘harmony’ and avoiding ‘inauspicious’ topics—leading nursing students and interns to exhibit avoidance behaviors when confronting death. Meanwhile, most students reported negative emotions when caring for dying patients, with low scores. For example, 33.50% of students agreed with item 7, ‘Caring for dying patients for long periods makes me feel very frustrated,’ and over 60.00% agreed with item 8, ‘I feel very sad when the dying patient I am responsible for loses hope for recovery’ (see Table 2). This high proportion of negative emotional responses suggests that nursing students may lack effective emotion regulation strategies when providing end-of-life care, providing a clear target for educational intervention.

Regression analysis revealed that palliative care was significantly associated with demographic characteristics such as gender, grade, attitude toward the nursing major, hospital internship experience, a family member with a terminal illness, previous bereavement experience, experience caring for dying patients, and previous lectures and training in palliative care. Internship experience and training experience were significantly associated with attitudes toward palliative care; however, the adjusted R² was only 0.084, indicating that these variables explained only a small portion of the variance in attitudes. Future studies should incorporate a broader range of relevant factors to improve the model’s explanatory power.

Regarding gender, there is no significant impact on attitudes [17,19], while others suggest that male students hold more positive attitudes toward palliative care than female students [22]. However, the findings of this study indicate that female students demonstrate more positive attitudes toward caring for dying patients compared to male students (*B* = 3.295*, P* < 0.001), which is consistent with the conclusions of several scholars [19,35,36]. This may be related to the empathy traits of females and their greater capacity for compassionate care [37], as women tend to exhibit stronger empathy toward dying patients and are more inclined to provide assistance. Notably, contrary to previous studies [17,19,37], this study found that nursing students in lower academic years held more positive attitudes toward palliative care. This might be because students in higher academic years experience a stronger fear of death [38], which may contribute to their less positive attitudes toward palliative care. In contrast to the findings of previous scholars [39], this study identified attitude toward the nursing profession as a significant factor associated with attitudes toward palliative care (*B* = −1.980*, P* = 0.049). From the perspective of Social Cognitive Theory, this finding can be understood through the fact that attitude is closely linked to the internalization of professional knowledge. Students with a positive attitude toward the nursing profession scored significantly higher on palliative care attitudes compared to those with a neutral attitude. Specifically, a stronger preference for the nursing profession was associated with more positive attitudes toward palliative care. This may be attributed to the fact that attitude is closely linked to the internalization of professional knowledge. Specifically, students who enjoy nursing tend to be more proactive in their learning and possess greater awareness of palliative care, thereby demonstrating more positive attitudes toward end-of-life care.

Personal experiences are widely recognized as an important factor associated with individuals’ attitudes, beliefs, and worldviews; accordingly, the relationship between different personal experiences and attitudes toward palliative care varies. Contrary to some research findings, internship experiences may subject students to significant moral and ethical pressure, leading to decreased confidence and perceived competence, with attitudes toward palliative care becoming less positive than before clinical placement [40–42]. However, this does not negate evidence suggesting that nursing students consider clinical internships the most important and effective approach to learning how to care for dying patients [20]. The present study found that nursing students with hospital internship experience held more positive attitudes toward palliative care (*B =* −1.980*, P* = 0.049). From the perspective of Social Cognitive Theory, clinical internships provide nursing students with opportunities for observational learning, hands-on practice, and vicarious experiences [43]. These experiences may effectively reduce students’ anxiety toward death-related situations and enhance their self-efficacy. Following direct clinical exposure, students appear to shift from initial uncertainty, anxiety, and fear toward greater confidence and develop deeper compassion for the patients they care for [44], resulting in more favorable attitudes toward end-of-life care. Therefore, incorporating increased hospital internship experiences into future curricula may be a promising approach to support more positive attitudes. Consistent with the findings of some authors [15,22], students who had a family member with a serious illness or experience caring for a relative tended to hold positive attitudes toward palliative care (*B =* 1.901*, P* = 0.038), possibly due to higher identification with end-of-life care arising from the need to care for a loved one.

However, regarding experiences of witnessing the death of a close family member or patient, this study reached conclusions contrary to those of some scholars. Researchers such as Gelegjamts D. and colleagues reported that such experiences were associated with positive attitudes [18,45]. In contrast, our study revealed that students who had experienced the death of a close family member exhibited more negative attitudes toward palliative care (*B* = −1.787*, P* = 0.042). From a theoretical perspective, Terror Management Theory (TMT) can explain this apparent paradox: unresolved bereavement experiences may activate or reinforce an individual’s deep-seated fear of death [31,46], and if this anxiety is not effectively channeled, it may instead induce avoidance behaviors. Second, an individual’s coping strategies when facing stressful events are critically important. Research has shown that the stress-coping patterns of nursing students during clinical internships may vary across different cultural and educational contexts [47]. If students lack structured grief counseling or meaning-making support, they may tend to adopt emotion-focused rather than problem-focused coping strategies, manifesting as negative attitudes toward palliative care. Therefore, a higher frequency of caring for terminally ill patients does not necessarily reduce death-related fear (*B* = −3.461*, P* = 0.039) – a finding that diverges from previous studies [22,48] – but precisely highlights the moderating role of coping mechanisms and support systems. Exposure to dying patients may evoke prior bereavement experiences and trigger stress and anxiety [49]. Consequently, if students receive psychological support and professional guidance from instructors, peers, or family members during bereavement or while caring for dying patients—such as through structured grief counseling or palliative care simulation training—this “isolated negative experience” may be transformed into a positive cognition toward palliative care.

In contrast, previous lectures and training in palliative care show a significant association with nursing students’ attitudes toward end-of-life care (*B* = −3.758*, P <* 0.001) [18–20], and it is linked to adopting a more open attitude toward caring for dying patients [50]. This is particularly important for nursing students interning in ICU, oncology departments, or emergency departments [15], as they need to correctly understand the meaning of palliative care and develop more positive attitudes, thereby enabling nursing students to actively engage in palliative care in their future nursing practice. Furthermore, studies have indicated that nursing students with bereavement experiences, cared for a dying patient, and positive attitudes toward palliative care demonstrate a greater need for death education or palliative care training [33]. Therefore, it is essential to systematically develop and refine palliative care education programs for nursing students, aiming to address critical gaps in death-coping abilities and mitigate the pervasive negative emotions associated with end-of-life care.

### Limitations

This study has several limitations. Although this study provides valuable insights into the attitudes of undergraduate nursing students toward palliative care, several limitations need to be considered. First, this study relied solely on quantitative survey data and did not explore the underlying reasons for changes in attitudes associated with different personal experiences. Moreover, the cross-sectional design does not allow for causal inferences. Future research should incorporate further qualitative studies and adopt longitudinal designs to clarify the direction of causality, which would contribute to a deeper understanding of the mechanisms and pathways through which nursing students’ personal experiences are associated with their attitudes toward palliative care. Second, the regression model in this study may have low explanatory power and included only a limited set of variables. This suggests the possible presence of omitted variable bias. For example, psychological traits of students (such as empathy, death anxiety, and self-efficacy) or prior educational quality may be associated with attitudes, but these variables may not have been fully included in the analysis given our focus on exploring the impact of personal experiences on attitudes toward palliative care. Therefore, future research should incorporate a more comprehensive range of potential associated factors to improve the explanatory power of the model and the robustness of the conclusions. Third, this study employed convenience sampling at a university in one province of China, which may introduce certain selection biases, and the results may not be generally applicable. The use of self-reported data may also introduce social desirability bias, particularly when addressing sensitive topics such as attitudes toward death. Nevertheless, despite these limitations, these results may still provide a reference for educators. Future research should conduct multicenter studies involving students from different regions to more comprehensively assess whether personally relevant experiences are associated with attitudes toward palliative care.

## Conclusions

This study indicates that undergraduate nursing students hold relatively positive attitudes toward palliative care. Specifically, previous lectures and training in palliative care were identified as factors positively associated with students’ attitudes. It is recommended that nursing schools integrate palliative care content into their compulsory curricula. Furthermore, theory should be integrated with practice. Nursing schools should strengthen cooperation with clinical practice sites to ensure that students have opportunities to encounter dying patients during their internships, along with targeted guidance from clinical instructors. A positive attitude toward the nursing profession is an important factor associated with more favorable attitudes toward palliative care. It is recommended that professional identity development modules be added to nursing curricula—through role modeling, reflective writing, and case discussions—to help students establish a positive professional identity, thereby indirectly enhancing their acceptance of palliative care.

Conversely, greater attention should be paid to students in higher academic years and those who have previous bereavement experience, as well as those who have cared for dying patients, as these factors were associated with less positive attitudes toward palliative care. Nursing educators should focus more on students’ emotional needs, strengthen death education and palliative care training, and ultimately cultivate highly competent nursing professionals for the field of end-of-life care.

### Data sharing and privacy protection statement

We confirm that no data will be shared in a manner that violates participant privacy. Identifiable information (e.g., names, addresses, IP addresses, specific dates, contact details, location data, or identifiable ID numbers) will not be disclosed. Furthermore, data from small or vulnerable groups that could lead to participant identification through indirect identifiers (e.g., gender, ethnicity, location) will also not be shared. All data presented have been anonymized and aggregated to protect participant confidentiality.

## Supporting information

S1 Table Demographic and experiential characteristics of nursing students (N = 467). (DOCX)
