# Supplementary material for: Factors associated with palliative care attitudes among undergraduate nursing students through personal experiences: A cross-sectional study
Source: PLoS One. 2026 Aug 14;21(8):e0355951. doi: 10.1371/journal.pone.0355951 (PMC13475884; doi:10.1371/journal.pone.0355951)
Supplement: Supplementary file 1 [file pone.0355951.s001.docx]

Table

**Table 1.** **Demographic and experiential characteristics of nursing students (N = 467).**

| **Students' characteristics** | **n(%)** | **The Attitude score of FATCOD-B-C** | **t/F** | **P** |
| --- | --- | --- | --- | --- |
| **Gender** |  |  |  |  |
| Male | 106(22.70) | 94.32±8.47 | -3.264 | 0.001 |
| Female | 361(77.30) | 97.46±8.77 |  |  |
| **Age** |  |  |  |  |
| 18-20 | 369(79.00) | 96.69±8.60 | -0.294 | 0.769 |
| 21-23 | 98(21.00) | 96.98±9.53 |  |  |
| **Nationality** |  |  |  |  |
| The Han nationality | 414(88.70) | 96.85±8.65 | -0.706 | 0.48 |
| Minority | 53(11.30) | 95.94±9.89 |  |  |
| **Religious belief** |  |  |  |  |
| No | 448(95.90) | 96.87±8.82 | 1.472 | 0.142 |
| Yes | 19(4.10) | 93.84±7.75 |  |  |
| **Residence** |  |  |  |  |
| Province-level region | 57(12.20) | 95.67±9.10 | 0.524 | 0.666 |
| Prefecture-level region | 61(13.10) | 96.28±8.34 |  |  |
| Town | 134(28.70) | 97.30±8.88 |  |  |
| Rural area | 215(46.00) | 96.82±8.82 |  |  |
| **Annual family income (RMB)** | | | | |
| ＜50000 | 221(47.30) | 95.98±8.71 | 1.129 | 0.337 |
| 50000-100000 | 161(34.50) | 97.51±8.44 |  |  |
| 100000-300000 | 75(16.10) | 97.44±9.02 |  |  |
| ＞300000 | 10(2.10) | 96.2±13.60 |  |  |
| **Self-rated health status** |  |  |  |  |
| Good | 325(69.60) | 96.77±8.61 | 0.008 | 0.992 |
| General | 123(26.30) | 96.72±9.42 |  |  |
| Unhealthy | 19(4.10) | 96.53±8.13 |  |  |
| **Grade** |  |  |  |  |
| Freshman | 180(38.50) | 97.24±8.54 | 1.176 | 0.319 |
| Sophomore | 100(21.40) | 95.32±8.12 |  |  |
| Junior | 110(23.60) | 97.23±8.74 |  |  |
| Senior | 77(16.50) | 96.77±10.18 |  |  |
| **Attitude toward nursing major** | | | | |
| Positive | 105(22.50) | 98.33±9.34 | 2.547 | 0.079 |
| Neutral | 327(70.00) | 96.41±8.54 |  |  |
| Negative | 35(7.50) | 95.14±9.09 |  |  |
| **Personal experiences** | | |  |  |
| **Hospital internship experience** | | | | |
| Yes | 88(18.80) | 97.32±10.11 | 0.605 | 0.546 |
| No | 379(81.20) | 96.62±8.47 |  |  |
| **Experience of participating in medical related voluntary service** | | |  |  |
| No | 278(59.50) | 96.49±8.36 | -0.752 | 0.453 |
| Yes | 189(40.50) | 97.13±9.41 |  |  |
| **Experience of suffering from serious illness** | | |  |  |
| No | 429(91.90) | 96.67±8.63 | -0.627 | 0.531 |
| Yes | 38(8.10) | 97.61±10.57 |  |  |
| **A family member with a terminal illness** | | |  |  |
| No | 319(68.30) | 96.37±8.74 | -1.363 | 0.147 |
| Yes | 148(31.70) | 97.56±8.89 |  |  |
| **Previous bereavement experience** | | |  |  |
| No | 210(45.00) | 97.57±9.48 | 1.835 | 0.067 |
| Yes | 257(55.00) | 96.07±8.16 |  |  |
| **History of contact with terminally ill patients** | | |  |  |
| No | 328(70.20) | 96.65±8.60 | -0.369 | 0.712 |
| Yes | 139(29.80) | 96.98±9.27 |  |  |
| **Experience of dealing with dead patients** | | |  |  |
| No | 414(88.70) | 96.96±8.90 | 1.438 | 0.151 |
| Yes | 53(11.30) | 95.11±7.85 |  |  |
| **Attendance at funerals** |  |  |  |  |
| No | 82(17.60) | 96.68±11.40 | 0.059 | 0.953 |
| Yes | 385(82.40) | 96.76±8.15 |  |  |
| **Cared for a dying patient** |  |  |  |  |
| No | 429(91.90) | 97.01±8.80 | 2.21 | 0.028 |
| Yes | 38(8.10) | 93.74±8.26 |  |  |
| **Previous lectures and training in palliative care** | | |  |  |
| Yes | 228(48.80) | 98.50±8.93 | 4.294 | ＜0.001 |
| No | 239(51.20) | 95.07±8.34 |  |  |

**Table 2. The results of the Chinese version of FATCOD-B-C (N = 467).**

***Reverse scored**

| **Item** | **Mean**  **(SD)** | **Strongly Agree**  **n(%)** | **Agree**  **n(%)** | **Uncertain**  **n(%)** | **Disagree**  **n(%)** | **Strongly Disagree**  **n(%)** |
| --- | --- | --- | --- | --- | --- | --- |
| 1. Giving care to the dying person is a worthwhile experience. | 4.51±0.78 | 293(62.70) | 139(29.80) | 23(4.90) | 4(0.90) | 8(1.70) |
| 2. Death is not the worst thing that can happen to a person. | 4.14±0.94 | 195(41.80) | 185(39.60) | 56(12.00) | 21(4.50) | 10(2.10) |
| 3. I would be uncomfortable talking about impending death with the dying person. ^*^ | 2.61±1.16 | 97(20.80) | 128(27.40) | 122(26.10) | 99(21.20) | 21(4.50) |
| 4. Caring for the patient's family should continue throughout the period of grief and bereavement. | 4.24±0.89 | 217(46.50) | 175(37.50) | 53(11.30) | 15(3.20) | 7(1.50) |
| 5. I would not want to care for a dying person. ^*^ | 3.26±1.09 | 41(8.80) | 54(11.60) | 168(36.00) | 150(32.10) | 54(11.60) |
| 6. The non-family care-givers should not be the one to talk about death with the dying person. ^*^ | 2.95±1.15 | 64(13.70) | 102(21.80) | 124(26.60) | 149(31.90) | 28(6.00) |
| 7. The length of time required to give care to a dying person would frustrate me. ^*^ | 2.95±1.04 | 39(8.40) | 117(25.10) | 171(36.60) | 109(23.30) | 31(6.60) |
| 8. I would be upset when the dying person I was caring for gave up hope of getting better. ^*^ | 2.33±0.99 | 87(18.60) | 213(45.6) | 105(22.5) | 48(10.3) | 14(3.0) |
| 9. It is difficult to form a close relationship with the dying person. ^*^ | 2.86±1.00 | 47(10.10) | 109(23.30) | 193(41.30) | 97(20.80) | 21(4.50) |
| 11. When a patient asks, "Am I dying?", I think it is best to change the subject to something cheerful. ^*^ | 2.37±0.95 | 86(18.40) | 187(40.00) | 137(29.30) | 50(10.70) | 7(1.50) |
| 12. The family should be involved in the physical care of the dying person if they  want to.^*^ | 4.09±0.82 | 145(31.00) | 246(52.70) | 58(12.40) | 10(2.10) | 8(1.70) |
| 13. I would hope the person I'm caring for dies when I am not present. ^*^ | 2.86±1.05 | 58(12.40) | 96(20.60) | 190(40.70) | 98(21.00) | 25(5.40) |
| 14. I am afraid to become friends with a dying person. ^*^ | 3.03±1.07 | 44(9.40) | 96(20.60) | 159(34.00) | 138(29.60) | 30(6.40) |
| 15. I would feel like running away when the person actually died. ^*^ | 3.15±1.01 | 37(7.90) | 65(13.90) | 190(40.70) | 141(30.20) | 34(7.30) |
| 16. Families need emotional support to accept the behavior changes of the dying person. | 3.94±0.88 | 127(27.20) | 220(47.10) | 95(20.30) | 17(3.60) | 8(1.70) |
| 17. As a patient nears death, the nonfamily caregiver should withdraw his/her involvement with the patient.^*^ | 1.88±0.87 | 170(36.40) | 209(44.80) | 67(14.30) | 14(3.00) | 7(1.50) |
| 18. Families should be concerned about helping their dying member make the best of his/her remaining life. | 4.20±0.83 | 188(40.30) | 210(45.00) | 52(11.10) | 10(2.10) | 7(1.50) |
| 19. The dying person should not be allowed to make decisions about his/her physical care.^*^ | 3.38±1.16 | 40(8.60) | 64(13.70) | 115(24.60) | 174(37.30) | 74(15.80) |
| 20. Families should maintain as normal an environment as possible for their dying member. | 4.02±0.85 | 141(30.20) | 221(47.30) | 86(18.40) | 13(2.80) | 6(1.30) |
| 21. It is beneficial for the dying person to verbalize his/her feelings. | 3.99±0.87 | 136(29.10) | 220(47.10) | 90(19.30) | 13(2.80) | 8(1.70) |
| 22. Care should extend to the family of the dying person. | 3.58±1.03 | 84(18.00) | 188(40.30) | 125(26.80) | 53(11.30) | 17(3.60) |
| 23. Care-givers should permit dying persons to have flexible visiting schedules. | 3.85±0.89 | 108(23.10) | 220(47.10) | 106(22.70) | 28(6.00) | 5(1.10) |
| 24. The dying person and his/her family should be the in-charge decision makers. | 3.61±1.01 | 96(20.60) | 171(36.60) | 131(28.10) | 60(12.80) | 9(1.90) |
| 25. Addiction to pain relieving medication should not be a concern when dealing with a dying person. | 2.91±1.19 | 48(10.30) | 108(23.10) | 118(25.30) | 138(29.60) | 55(11.80) |
| 26. I would be uncomfortable if I entered the room of a terminally ill person and found him/her crying. ^*^ | 2.46±0.96 | 63(13.50) | 204(43.70) | 138(29.60) | 46(9.90) | 16(3.40) |
| 27. Dying persons should be given honest answers about their condition. | 3.35±0.89 | 51(10.90) | 136(29.10) | 214(45.80) | 58(12.40) | 8(1.70) |
| 28. Educating families about death and dying is not a non-family care-givers responsibility.^*^ | 3.35±1.17 | 42(9.00) | 72(15.40) | 102(21.80) | 181(38.80) | 70(15.00) |
| 29. Family members who stay close to a dying person often interfere with the  professionals' job with the patient. | 2.94±1.01 | 45(9.60) | 93(19.90) | 202(43.30) | 100(21.40) | 27(5.80) |
| 30. It is possible for non-family care-givers to help patients prepare for death. | 3.91±1.01 | 106(22.70) | 239(51.20) | 102(21.80) | 13(2.80) | 7(1.50) |

**Table 3. Assignment Table.**

| **Correlation variables** | **Assignment Instructions** |
| --- | --- |
| Gender | Male = 1; Female = 2 |
| Age | 18-20 = 1; 21-23 = 2 |
| Nationality | The Han nationality = 1; Minority = 2 |
| Religious belief | No = 1; Yes = 2 |
| Self-rated health status | Good = 1; General = 2; Unhealthy = 3 |
| Grade | Freshman = 1; Sophomore = 2; Junior = 3; Senior = 4 |
| Attitude toward nursing major | Positive = 1; Neutral = 2; Negative = 3 |
| **Personal experiences** | |
| Hospital internship experience | Yes = 1; No = 2 |
| Experience of participating in medical related voluntary service | No = 1; Yes = 2 |
| Experience of suffering from serious illness | No = 1; Yes = 2 |
| A family member with a terminal illness | No = 1; Yes = 2 |
| Previous bereavement experience | No = 1; Yes = 2 |
| History of contact with terminally ill patients | No = 1; Yes = 2 |
| Experience of dealing with dead patients | No = 1; Yes = 2 |
| Attendance at funerals | No = 1; Yes = 2 |
| Cared for a dying patient | No = 1; Yes = 2 |
| Previous lectures & training in palliative care | Yes = 1; No = 2 |

**Table 4. Multiple Linear Regression Analysis of FATCOD-B-C Scores among Undergraduate Nursing Students (N = 467).**

| **Variable** | **B** | **SE** | **β** | **t** | ***P*** | **95% CI** | |
| --- | --- | --- | --- | --- | --- | --- | --- |
|  |  |  |  |  |  | **Lower bound** | **Upper bound** |
| Constant | 110.782 | 6.774 | - | 16.354 | ＜.001 | 97.469 | 124.096 |
| Gender=Female  (Ref: Male) | 3.295 | 0.974 | 0.157 | 3.382 | ＜.001 | 1.380 | 5.210 |
| Grade=Sophomore  (Ref: Freshman) | -2.233 | 1.124 | -0.104 | -1.988 | .047 | -4.442 | -0.025 |
| Grade = Junior  (Ref: Freshman) | -3.037 | 1.29 | -0.147 | -2.355 | .019 | -5.572 | -0.503 |
| Grade = Senior  (Ref: Freshman) | -8.626 | 3.591 | -0.364 | -2.402 | .017 | -15.684 | -1.568 |
| Attitude toward nursing major = Neutral  (Ref: Positive) | -1.980 | 1.003 | -0.103 | -1.975 | .049 | -3.951 | -0.010 |
| Hospital internship experience (Ref: Yes) | -5.362 | 2.713 | -0.239 | -1.976 | .049 | -10.695 | -0.030 |
| A family member with a terminal illness (Ref: No) | 1.901 | 0.912 | 0.101 | 2.084 | .038 | 0.108 | 3.693 |
| Previous bereavement experience (Ref: No) | -1.787 | 0.875 | -0.101 | -2.042 | .042 | -3.507 | -0.067 |
| Cared for a dying patient (Ref: No) | -3.461 | 1.672 | -0.108 | -2.07 | .039 | -6.747 | -0.175 |
| Previous lectures and training in palliative care (Ref: Yes) | -3.758 | 0.923 | -0.214 | -4.072 | ＜.001 | -5.572 | -1.944 |

Note: *B*, regression coefficient; *SE,* Standard error; *Beta*,Standardized coefficient Beta

F = 2.581, P < .001, R = 0.370, coefficient of determination (R^2^) = 0.137, adjusted R^2^= 0.084
